# UGT2B17 copy number gain in a large ankylosing spondylitis multiplex family

**DOI:** 10.1186/1471-2156-14-67

**Published:** 2013-08-08

**Authors:** Mohammed Uddin, Walter P Maksymowych, Robert Inman, Dafna Gladman, Alexandra Munn, Ramin Yazdani, Fawnda Pellett, Sean Hamilton, Darren D O’Rielly, Proton Rahman

**Affiliations:** 1 Faculty of Medicine, Memorial University of Newfoundland, St. John’s, NF, Canada; 2 Department of Medicine, University of Alberta, Edmonton, AB, Canada; 3 Faculty of Medicine, University of Toronto, Toronto, ON, Canada

**Keywords:** Copy number variants (CNV), UGT2B17, Ankylosing spondylitis, Complex autoimmune disease

## Abstract

**Background:**

The primary objective of this study is to identify novel copy number variations (CNVs) associated with familial ankylosing spondylitis (AS). A customized genome-wide microarray was designed to detect CNVs and applied to a multiplex AS family with six (6) affected family members. CNVs were detected using the built-in DNA analytics aberration detection method-2 (ADM-2) algorithm. Gene enrichment analysis was performed to observe the segregation. Subsequent validation was performed using real time quantitative fluorescence polymerase reaction (QF-PCR). The frequency of copy number variation for the UGT2B17 gene was then performed on two well-defined AS cohorts. Fisher exact test was performed to quantify the association.

**Results:**

Our family-based analysis revealed ten gene-enriched CNVs that segregate with all six family members affected with AS. Based on the proposed function and the polymorphic nature of the UGT2B17 gene, the UGT2B17 gene CNV was selected for validation using real time QF-PCR with full concordance. The frequency of two copies of the UGT2B17 gene CNV was 0.41 in the Newfoundland AS cases and 0.35 in the Newfoundland controls (OR = 1.26(0.99-1.59); p < 0.05)), whereas the frequency of two (2) copies of the UGT2B17 gene CNV was 0.40 in the Alberta AS cases and 0.39 in the Alberta controls (OR = 1.05(95% CI: 0.83-1.33); p < 0.71)).

**Conclusions:**

A genome-wide microarray interrogation of a large multiplex AS family revealed segregation of the UGT2B17 gene CNV among all affected family members. The association of the UGT2B17 CNV with AS is particularly interesting given the recent association of this CNV with osteoporosis and the proposed function as it encodes a key enzyme that inhibits androgens. However, two copies of the UGT2B17 gene CNV were only marginally significant in a uniplex AS cohort from Newfoundland but not in a uniplex AS cohort from Alberta.

## Background

Ankylosing spondylitis (AS) is the prototypic inflammatory spondyloarthritis with a peak onset between 20 to 30 years [1,2]. Extra-articular features of AS include inflammation of the eyes, skin, bowels, and more rarely the lungs and heart. There is a male preponderance as men are affected 2 to 3 times more frequently than females [3,4].

Genetic factors are of major importance in susceptibility to AS and, in fact, genetic epidemiological studies suggest that AS represents one of the most heritable complex autoimmune diseases with a heritability greater than 90% and a sibling recurrence ratio of at least 52 [2-4]. HLA-B*27, which was first recognized to be associated with AS in 1973, remains the strongest genetic association signal with AS, and it is estimated that HLA-B*27 accounts for 23% of the genetic heritability [2-4]. Despite this strong association, only a small fraction (1-5%) of HLA-B*27 positive individuals develop AS [2-4]. Recent genome-wide association studies (GWAS) in several European populations and in the Han Chinese population have identified up to 15 high priority genes including IL23R, RUNX3, KIF21B, 2p15, IL1R2, PTGER4, ERAP1, IL12B, CARD9, TNFR1/ LTBR, TBKBP1 [5]. These associations were primarily reported based on single nucleotide polymorphisms (SNP) analysis. Recently, an association study revealed the complex interactions between the non-HLA gene, ERAP1, with the HLA-B*27 gene [5,6]. However, the genetic risk described by HLA or non-HLA genes suggests that other genomic variants may contribute to the risk factor for AS.

The premise that CNVs contribute to disease pathogenesis is supported by their capacity to disrupt gene expression and to interrupt functional pathways [7]. In recent years, many CNVs have been associated with complex diseases and the majority of these associations were in autoimmune-mediated diseases, including rheumatoid arthritis, psoriasis, Crohn’s disease, and systemic lupus erythematous [8-11]. Here, we report the first validated CNV associated specifically with AS using a custom genome-wide microarray in a well-defined multiplex AS family.

## Results

### Multiplex family

**Figure 1:**
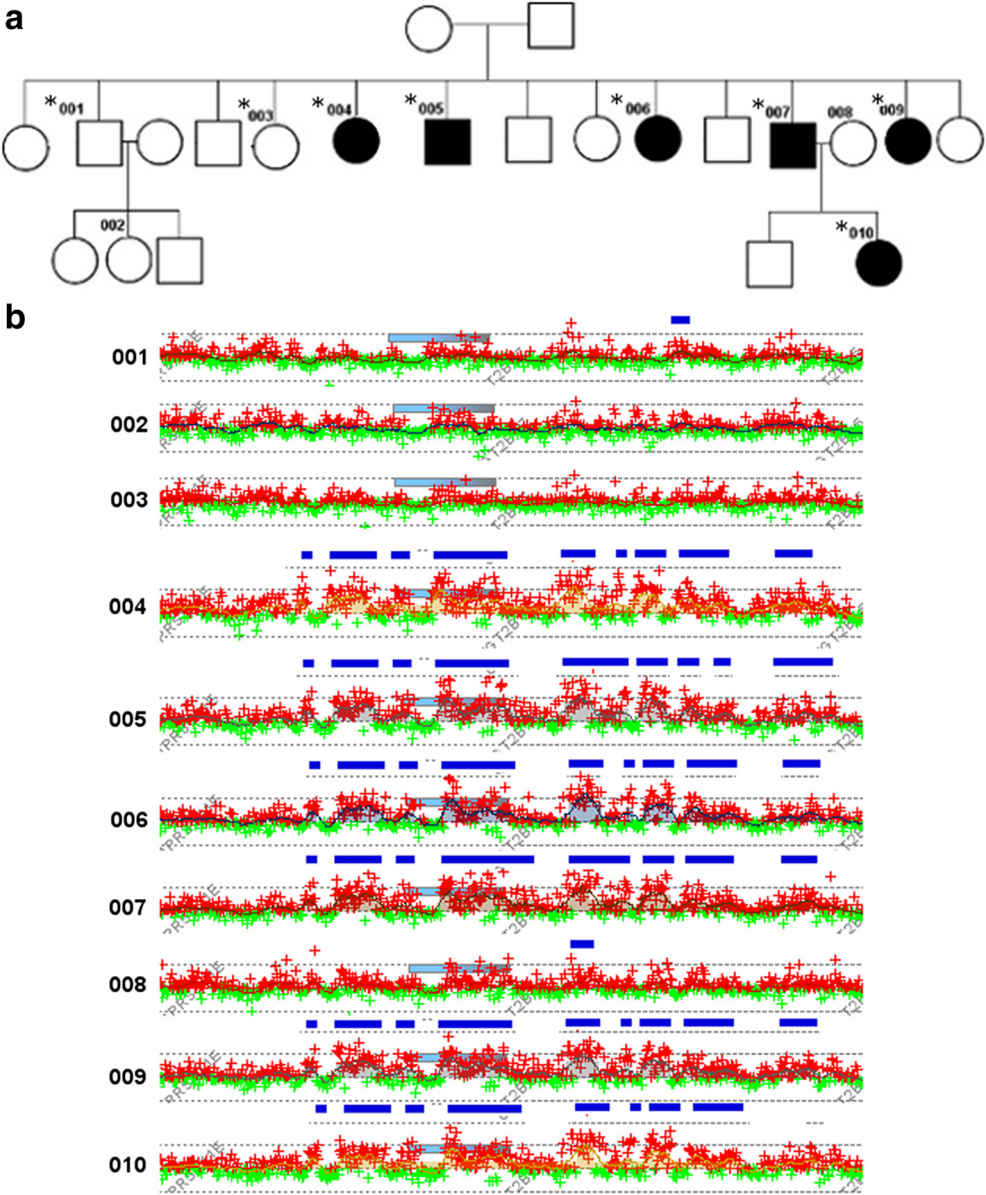
**a)A three generational pedigree comprising 13 individuals.** DNA was available only for the individuals with an identification number (Ids). The affected family members are indicated by solid colors and member 002 has systemic lupus erythematous (SLE). **b)** The microarray intensity values for the UGT2B17 region are indicated below the pedigree for each of the family members. Each green/red point represents the probe intensities, the cyan rectangle represents the gene location, and horizontal dark blue rectangles represent duplications detected by the genome-wide analysis of CNVs.

**Figure 2:**
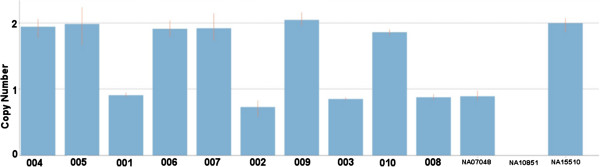
**UGT2B17 gene copy number validation using real time QF-PCR on family members and three reference HapMap samples.**

The quality control (QC) measures for the custom microarray chip were very good as all samples exhibited <0.25 DLRS. Approximately 1700 CNVs were detected in each individual sample using our custom microarray. Segregated gene-centric CNV analysis (i.e., a CNV that consists of or overlaps with a gene) revealed that 56 CNVs are enriched in affected family members (at least three) and absent in the unaffected family members. Among these 56 CNVs, we have identified ten (9) gene copy number variation regions (CNVRs) that segregate with at least six (6) affected family members (Additional file 1: Table S1). Our microarray analysis revealed multiple duplications within the UGT2B17 gene region that segregate in the affected family members that is absent in unaffected family members. One 7 kb duplication (chr4:69423002–69430016) disrupts exon 3 of the UGT2B17 gene and segregates with six AS affected family members (Figure 1). The breakpoint encompassing the UGT2B17 gene region was covered with probes with 280 bp spacing, providing high resolution to detect genomic aberrations. Validation of this targeted region using real time QF-PCR demonstrated 100% concordance with the microarray analysis (Figure 2). The real time QF-PCR CNV call (>99% confidence) revealed that affected family members carried two (2) copies of the UGT2B17 gene, whereas unaffected members carried a single copy. Eight (8) of the ten (10) family members were HLA-B27 positive including and all six affected family members that carried two (2) copies of the UGT2B17 gene.

### Disease prevalence (case control studies)

In the case–control cohorts, real time QF-PCR analysis targeting the identical CNVR (from family analysis) revealed the presence of three (3) alleles (0 copy - homozygous deletion, 1 copy - heterozygous deletion, and 2 copies) within this population for the UGT2B17 gene.

In the Newfoundland population, the frequency of the three alleles was 0.13, 0.51, and 0.35 for 0, 1 and 2 copies in controls, respectively. The frequency of two (2) copies of the UGT2B17 gene CNV was 0.41 in the Newfoundland AS cases and 0.35 in the Newfoundland controls (OR = 1.26 (95% CI:0.99-1.59); p = 0.05). The frequency of two (2) copies of the UGT2B17 gene in the Alberta population was 0.40 and 0.39 for AS cases and controls, respectively (OR = 1.05 (95% CI:0.83-1.33); p = 0.71) (Table 1). The association analysis for the combined cohort (Newfoundland and Alberta population) revealed two (2) copies of the UGT2B17 gene with a frequency of 0.40 and 0.37, for the cases and controls, respectively (association significance p = 0.09).

**Table 1:** UGT2B17 gene CNV association in ankylosing spondylitis

**Population**	**Copy numbers**	**AS cases**	**Healthy controls**	**OR(95%CI)**	**P value**
**Newfoundland**	2	0.41	0.35	1.26(0.99-1.59)	<0.05
298 cases					
299 controls					
**Alberta**	2	0.40	0.39	1.05(0.83-1.33)	<0.71
289 cases					
285 controls					

## Discussion

Copy number variations are increasingly being recognized in complex autoimmune diseases as they are capable of altering gene dosage and consequently affect gene function [12]. The contribution of CNVs in AS pathogenesis remains to be systematically evaluated. The primary goal of this study was to identify functionally relevant CNVs segregating within a multiplex AS family and then subsequently determine the allele frequencies of CNVs in multiple case–control cohorts. The genome-wide CNV analysis performed in this study revealed that increased copy number of the UGT2B17 gene is a potential risk factor for AS.

Given that UGT2B17 is a functional gene encoding an enzyme that metabolizes steroid hormones including testosterone and selected xenobiotics [13-16], it represents an excellent candidate gene for susceptibility to AS. A gene-dosage effect is present as this CNV is associated with urine testosterone level, male insulin sensitivity, fat mass, and prostate-cancer risk (as summarized by Xue) [13]. Importantly, copies of the UGT2B17 gene have recently been associated with osteoporosis (including hip fracture) [14]. The dose of the UGT2B17 gene was significantly associated with bone mineral density, cortical thickness, and buckling ratio. These results support the role of UGT2B17 CNVs in the pathogenesis of osteoporosis. Although AS is characterized by new bone formation, osteoporosis is a well recognized complication of AS and the risk of clinical vertebral fractures is increased in AS patients [17,18]. This complexity with respect to genotype-phenotype correlation requires further investigation.

From an evolutionary perspective, the UGT2B17 gene in humans is highly stratified which renders it more likely to be associated with disease. The deletion allele (i.e., 0 copies) frequency varies significantly among populations. For example, the frequency of the deletion allele is approximately 0.15 in Europeans, ~0.22 in Africans, and ~0.80-0.90 in Asians [7,13]. In contrast, the frequency of two (2) copies of the UGT2B17 gene is rare in Asian populations (~0.01-0.03), ~0.30-0.40 (reports with varying frequencies) in Europeans, and ~0.60 in African populations.

In this study, two copies of the UGT2B17 gene clearly segregates within a large well-defined AS multiplex family. Given the nature and proposed function of the UGT2B17 gene [19,20], the UGT2B17 CNV may contribute to susceptibility for AS within this family. Conflicting results were obtained regarding the excess prevalence of this CNV in large uniplex AS cohorts. A trend was observed in the cohort from Newfoundland. However, this was not replicated in the Alberta cohort. In this study, the deletion (i.e., 0 copies) frequency of the UGT2B17 gene in the Newfoundland population is consistent with the Alberta population. There was a noticeable frequency difference between the Alberta (0.51) and Newfoundland (0.46) populations for a single copy of the UGT2B17 gene. This stratification may represent a contributing factor for the lack of association.

The analysis of CNVs within multiplex families provides a unique opportunity to detect variants that may be family-specific. There are multiple reports in other complex diseases where family specific CNVs manifest with the disease [21]. Although it is a difficult task to obtain more multi-generational pedigrees to follow up the segregation of this CNV, the findings observed within the investigated family suggests that segregation analysis into multiple multi-generation pedigrees is promising. Thus, it is important to assess other large multiplex families to determine if this CNV may be a cause of familial AS.

## Conclusions

This is study applied a custom designed genome-wide microarray to investigate a large multiplex AS family revealed segregation of the UGT2B17 gene CNV among all affected family members. The association of the UGT2B17 CNV with AS is particularly interesting given the recent association of this CNV with osteoporosis and the proposed function as it encodes a key enzyme that inhibits androgens. This result provides a basis for further exploration on the role of CNV in AS pathogenesis. Further replication is required for the marginal significance of UGT2B17 association identified with AS cohort from Newfoundland.

## Methods

### Multiplex AS family

A multiplex AS family from Newfoundland was identified for this study. All members of the multiplex family were “native Newfoundlanders” of North European ancestry. Ten (10) members from this large family participated in the study. Each individual was assessed clinically including imaging studies. Six (6) of the ten (10) family members had AS, as defined by the 1984 modified New York criteria, while the remaining family members were not affected. Informed consent for participation in the study was obtained from participants or, where participants are children, a parent or guardian. After consent was obtained, all participating family members had their DNA collected. All samples involving human subjects reported in this manuscript have been approved by the Memorial University of Newfoundland Human Investigation Committee.

### Case–control cohorts

DNA was obtained from two (2) well-defined AS cohorts from the Newfoundland and Alberta populations. All cases and controls were of North European ancestry. We assessed 298 AS cases satisfying the modified New York criteria and 299 ethnically-matched controls from a homogenous population of Newfoundland. The second cohort consisted of 289 AS cases satisfying the modified New York criteria and 285 ethnically-matched controls from Alberta.

### Custom microarray

We designed a customized genome-wide microarray based on genomic hotspot breakpoints previously identified [22]. The targeted regions are prone to produce CNVs due to their structural architecture (i.e. presence of segmental duplication, dose sensitive genes etc.). The custom design tiling microarray consisted of 2 × 1 million probes covering the genome with a mean spacing of 280 bp. Prior to CNV analysis, QC measures were applied and the derivative of log ratio spread (DLRS) <0.25 was considered as threshold. CNVs were detected using the built-in Aberration Detection Method-2 (ADM-2) algorithm DNA Analytics v.4.0.85 (Agilent Technologies) using the following criteria: 1) at least 5 probes for a CNV call on GC-corrected intensity; 2) nested filter was set to 2; and 3) log intensity >0.25 for duplication and <−0.25 for deletion. A custom script was applied to detect gene-enriched CNVs (i.e., overlaps with or constitutes a gene) that segregate (at least three cases) within affected AS family members and absent in the unaffected family members.

### QF-PCR

To validate the CNV encompassing the UGT2B17 (UDP glucuronosyltransferase 2 family, polypeptide B17) gene in the family members and in the case–control cohorts, copy number estimation of the UGT2B17 gene was performed using the Taqman copy number assay Hs03185327_cn (Life Technologies) using the manufacturer’s recommended protocol. The assay was performed in quadruplicate using 10ng genomic DNA for each sample in a 96-well plate. The 10 μl reaction mix consisted of 2 μl of 2× Taqman Genotyping Master Mix (Life Technologies), 0.5 μl of 20× copy number assay (described above), 0.5 μl of TaqMan RNAse P Copy Number Reference Assay (Life Technologies, part 4403326), 2 μl of water, and 2 μl of 5 ng/μl genomic DNA. Cycling conditions for the reaction were 95°C for 10 min, followed by 40 cycles of 95°C for 15 sec and 60°C for 1 min. Samples were analyzed using the ViiA™ 7 Real-Time PCR System (Life Technologies) and analyzed using CopyCaller Software (Life Technologies, PN 4412907). Three reference (calibrator) DNA HapMap samples (NA10851, NA15510 and NA07048 (Coriell Institute)) plus one non-template control were included with the test samples. Fisher exact test was performed to determine association of the UGT2B17 gene copy number with AS in our case–control data.

## Competing interests

The authors have declared that no competing interests exist.

## Authors’ contributions

MU, DO and PR conceived the problem and designed the project. MU implemented the methodology and carried out the analysis. WPM, RI, DG, AM, RY, FP, SH, and PR contributed on the patient recruitment. The manuscript was written by MU, DO, and PR. All authors read and approved the final manuscript.

## Supplementary Material

Additional file 1: Table S1 A list of gene-containing CNVs that segregate with six AS affected individuals within the family. Click here for file
