# Supplementary material for: UGT2B17 copy number gain in a large ankylosing spondylitis multiplex family
Source: BMC Genet. 2013 Aug 8;14:67. doi: 10.1186/1471-2156-14-67 (PMC3751806; doi:10.1186/1471-2156-14-67)
Supplement: Additional file 1: Table S1 — A list of gene-containing CNVs that segregate with six AS affected individuals within the family. [file 1471-2156-14-67-S1.docx]

**UGT2B17 Copy Number Gain in a Large Ankylosing Spondylitis Multiplex Family**

**Mohammed Uddin^1^, Robert Inman^2^, Walter Maksymowych^3^, Dafna Gladman^2^, Ramin Yazdani^1^, Fawnda Pellett^2^, Sean Hamilton^1^, Darren D. O’Rielly^1^, Proton Rahman^1,^ ***

# Additional file 1

**Additional file 1: Table S1.** A list of gene-containing CNVs that segregate with six AS affected individuals within the family.

| **Chr** | **Start** | **End** | **Length**  **(bp)** | **# of Probes** | **Status** | **Gene(s)** |
| --- | --- | --- | --- | --- | --- | --- |
| 4 | 69518934 | 69530196 | 11262 | 40 | gain | *UGT2B15* |
| 9 | 84533087 | 84558449 | 25362 | 56 | gain | *FLJ43950, FLJ43859, FLJ44082* |
| 10 | 48747724 | 49389683 | 641959 | 1857 | gain | *PTPN20B, PTPN20A, FRMPD2L1, FRMPD2L2, BMS1P5, BMS1P1, FAM25B, FAM25C, FAM25G, LOC399753, FRMPD2* |
| 14 | 36327173 | 36329122 | 1949 | 7 | loss | *BRMS1L* |
| 17 | 43589332 | 43597242 | 7910 | 27 | loss | *LRRC37A4* |
| 2 | 3440855 | 3441900 | 1045 | 6 | loss | *TTC15* |
| 5 | 677193 | 852102 | 174909 | 592 | loss | *TPPP, ZDHHC11* |
| 5 | 69534796 | 69559366 | 24570 | 86 | gain | *LOC653391* |
| 13 | 77871860 | 77874499 | 2639 | 10 | gain | *MYCBP2* |
